# Long-Term Effects of Sevelamer on Vascular Calcification, Arterial Stiffness, and Calcification Propensity in Patients Receiving Peritoneal Dialysis: The Randomized Pilot SERENE (Sevelamer on Vascular Calcification, Arterial Stiffness) Trial

**DOI:** 10.1016/j.xkme.2021.10.002

**Published:** 2021-11-02

**Authors:** Angela Yee-Moon Wang, Andreas Pasch, Chun-Kwok Wong, Ida Miu-Ting Chu, Tak-Ka Tang, Jessie Chu, Charmaine Cheuk-Ying Fong, Yat-Yin Yau, Wai-Kei Lo

**Affiliations:** 1 Department of Medicine, Queen Mary Hospital, The University of Hong Kong, Hong Kong SAR, China; 2 Institute for Physiology and Pathophysiology, Johannes Kepler University Linz, Linz, Austria; 3 Practice for Internal Medicine and Nephrology at Hirschengraben, Bern, Switzerland; 4 Calciscon AG, Biel, Switzerland; 5 Department of Nephrology, Lindenhofspital Bern and Nierenpraxis Bern, Bern, Switzerland Calciscon AG, Nidau, Switzerland; 6 Department of Chemical Pathology, Prince of Wales Hospital, Chinese University of Hong Kong, Hong Kong SAR, China; 7 Precision Health & Diagnostic Centre Ltd, Hong Kong SAR, China; 8 Department of Medicine, Tung Wah Hospital, The University of Hong Kong, Hong Kong SAR, China

**Keywords:** Aortic stiffness, calcification propensity, heart valve calcification, hyperphosphatemia, lipid lowering, non–calcium-containing, phosphorus binder, pulse wave velocity, sevelamer, vascular calcification

## Abstract

**Rationale & Objective:**

There is a concern regarding increased risk of vascular calcification with the use of calcium-based phosphorus binders. This study aimed to compare the effects of sevelamer used as a second-line, low-dose therapy with calcium-based phosphorus binders with those of sevelamer used as a first-line, high-dose therapy on coronary artery and heart valve calcification, aortic pulse wave velocity (PWV), and calcification propensity over 2 years in patients with hyperphosphatemia receiving peritoneal dialysis (PD).

**Study Design:**

A 2-year-long prospective, multicenter, open-label, randomized pilot study.

**Setting & Participants:**

Prevalent patients with hyperphosphatemia receiving PD from 2 university-affiliated hospitals in Hong Kong.

**Interventions:**

The patients were randomized to receive sevelamer either as a first-line therapy at a high dose of 800 mg thrice daily (can titrate up to 1,200 mg thrice daily as required) or a second-line therapy at a low dose of 400 mg thrice daily with calcium carbonate to achieve a serum phosphorus target of ≤5.5 mg/dL.

**Outcomes:**

The primary endpoints were changes in coronary artery calcium score and aortic PWV over 104 weeks. The secondary endpoints were changes in heart valve calcium scores, calcification propensity measure, and biochemical parameters of chronic kidney disease–mineral bone disease over 104 weeks.

**Results:**

Among 60 prevalent patients receiving PD, with a mean age of 53 ± 10 years and with 57% men, changes in the coronary artery calcium score (median [interquartile range], 225 [79-525] vs 223 [56-1,212], respectively; *P* = 0.21), aortic PWV (mean ± standard error, 0.3 ± 0.1 vs 0.8 ± 0.2 m/s, respectively; *P* = 0.31), heart valve calcium score, maturation or transformation time, serum calcium levels, and phosphorus levels over 104 weeks were similar for the second-line, low-dose and first-line, high-dose sevelamer groups. Alkaline phosphatase and intact parathyroid hormone levels increased and low-density lipoprotein cholesterol decreased in both the groups, with no significant between-group differences.

**Limitations:**

The sample size was small, and the dropout rates were relatively high.

**Conclusions:**

Low-dose sevelamer used as a second-line therapy for hyperphosphatemia in combination with a calcium-based phosphorus binder had similar effects on vascular calcification, valvular calcification, and arterial stiffness compared with high-dose sevelamer used as a first-line therapy. This approach may be considered in resource-constrained countries to minimize calcium loading.

**Funding:**

The study was supported by a competitive grant from SK Yee Medical Foundation. T50 assays and other biochemical assays were funded by a research grant from Sanofi Renal Corporation.

**Trial Registration:**

NCT00745589.

## Graphical abstract

**Figure:**
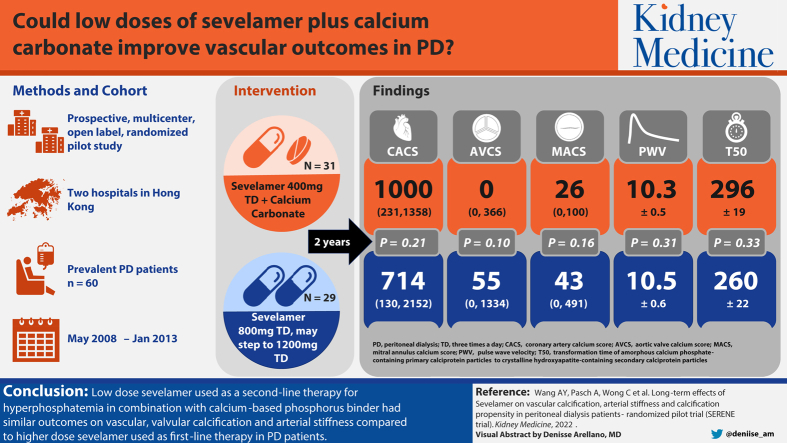

**Plain-Language Summary**

This is a randomized study aiming to compare the effects of sevelamer, a non–calcium-containing phosphorus binder, used as a second-line therapy at a low dose with a calcium-based phosphorus binder versus those of sevelamer used as a first-line therapy at a higher dose on heart blood vessels and heart valve calcification, arterial stiffness, and calcification propensity over 104 weeks in patients with kidney failure receiving peritoneal dialysis. The results showed that low-dose sevelamer used as a second-line therapy for hyperphosphatemia in combination with a calcium-based phosphorus binder had similar effects on heart vessels, heart valve calcification, and arterial stiffness as high-dose sevelamer used as a first-line therapy. This approach may be considered in resource-constrained countries to minimize calcium loading.

Vascular and valvular calcification is an important complication in patients receiving dialysis, and these help predict adverse clinical outcomes.^1^, ^2^, ^3^ Vascular calcification is frequently progressive, especially among those with baseline vascular calcification.^4^ Thus, limiting the progression of vascular or valvular calcification is an important therapeutic goal in patients receiving dialysis. Earlier randomized studies have suggested that sevelamer attenuates the progression of coronary and aortic calcification in patients receiving hemodialysis compared with calcium-based phosphorus binders.^4^^,^^5^ The use of sevelamer in patients with chronic kidney disease (CKD) 3-4 may lower the risk of death and dialysis compared with the use of calcium-based phosphorus binders.^6^ Two meta-analyses of randomized trials have suggested that non–calcium-based phosphorus binders reduce the risk of progression of coronary artery calcification compared with calcium-based phosphorus binders in patients with CKD.^7^^,^^8^ Another systematic review has suggested that although sevelamer lowers the all-cause death rate compared with calcium-based binders, no clinically important benefits of any phosphate binder were observed on cardiovascular deaths or coronary artery calcification.^9^ Additionally, the vascular effects of sevelamer in patients receiving peritoneal dialysis (PD) are limited. It is uncertain whether the progression of coronary artery calcification and arterial stiffness relate to calcification propensity and the dose of sevelamer used.

In uremic serum, crystalline hydroxyapatite is present as spindle-shaped, high-molecular-weight, protein-mineral complexes, termed as secondary calciprotein particles (CPPs). These secondary CPPs are derived from spherical amorphous calcium phosphate-containing primary CPPs and fetuin-A.^10^ Low serum fetuin-A levels have been shown to be associated with increased risk of mortality and adverse cardiovascular outcomes in patients with CKD.^11^^,^^12^ A nanoparticle-based test that measures the overall serum calcification propensity by monitoring the maturation or transformation time (T50) from primary CPPs to secondary CPPs in the serum showed reduced intrinsic properties to inhibit calcification in sera from mice deficient in fetuin-A and in sera from patients receiving hemodialysis.^13^ Clinically, a lower T50 has been shown to predict higher mortality risk in patients with CKD.^14^ A post hoc analysis from the Evaluation of Cinacalcet Hydrochloride Therapy to Lower Cardiovascular Events trial showed that a lower serum T50 predicted greater risk of all-cause mortality and cardiovascular events in patients receiving hemodialysis.^15^ A lower T50 has also been shown to be associated with a higher aortic pulse wave velocity (PWV) and predicts progressive aortic stiffening in patients with CKD.^14^ However, it is not known whether sevelamer may increase T50 in patients receiving PD.

We hypothesized that low-dose sevelamer, used as a second-line phosphorus binder along with calcium carbonate, may have similar effects as first-line, high-dose sevelamer therapy on coronary artery calcification, arterial stiffening, aortic valve and mitral annulus calcification, T50, and biochemical parameters of CKD–mineral bone disease (MBD) over a 2-year period in patients receiving PD.

## Methods

This is a prospective, multicenter, open-label, randomized pilot study of 104 weeks and is an investigator-initiated study. The study protocol was approved by the institutional research ethics board of the University of Hong Kong/Hong Kong West cluster of Hong Kong (UW07-090). The study was registered with ClinicalTrials.gov as NCT00745589. Sixty eligible patients with kidney failure receiving long-term PD were recruited from 2 university-affiliated hospitals, Queen Mary Hospital and Tung Wah Hospital of the University of Hong Kong, after they provided informed consent. A sample size calculation was not performed because this was a pilot study. Study participant recruitment was started in May 2008 and completed in January 2013. The eligibility criteria for the study participants included patients with kidney failure receiving long-term PD treatment, age between 25 and 80 years, patients receiving an aluminum-based phosphorus binder, patients with serum phosphorus levels >5.51 mg/dL (upper limit of laboratory reference range) despite receiving a calcium-based phosphorus binder, and an intact parathyroid hormone (iPTH) level of <857 pg/mL because an iPTH level of > 857 pg/mL would indicate referral for parathyroidectomy. The exclusion criteria included patients with an underlying malignancy or cyanotic congenital heart disease; patients with plans for undergoing a living-related kidney transplant within a year; patients who were ill, wheelchair-bound, or required assistance with activities of daily living; or female patients with a planned pregnancy.

Recruited patients were randomized to receive sevelamer hydrochloride as either a first-line therapy at a high dose or a second-line therapy at a low dose after the administration of calcium carbonate. Aluminum-based phosphorus binders were stopped at that time of study entry. This randomization was performed at the baseline follow-up visit by a study investigator (A.Y.-M.W.) by drawing lots. The study coordinator prepared all the lots and placed them in a dark-colored bag before selection.

For patients randomized to second-line, low-dose sevelamer, calcium carbonate was used as the first-line phosphorus binder and the serum calcium level was maintained at <10.3 mg/dL (upper limit of laboratory reference). If the serum phosphorus level remained at >5.51 mg/dL (upper limit of laboratory reference), a fixed dose of sevelamer hydrochloride of 400 mg was given thrice daily, but the calcium carbonate dose was not fixed. For patients randomized to the first-line, high-dose group, sevelamer at 800 mg thrice daily was given as the first-line treatment. The dose was uptitrated to 1,200 mg thrice daily, as required, to achieve a target serum phosphorus level of <5.51 mg/dL. Calcium carbonate could be used after sevelamer, but the serum calcium level had to be maintained at <9.50 mg/dL and the elemental calcium intake was not to exceed 1,500 mg daily. All patients were counseled by physicians every 3 months to reinforce dietary adherence to the phosphorus restriction.

### Clinical Data Collection

We collected data on smoking status, diabetes mellitus, coronary artery disease, heart failure, ischemic or hemorrhagic cerebrovascular events, Charlson comorbidity score, and medication use at the time of study entry. At each 12-week follow-up, systolic and diastolic blood pressures were measured using a sphygmomanometer twice on either arm after the patient had rested for 15 minutes, and the measurements were then averaged.

### Multislice Computed Tomography of Coronary Arteries and Heart Valve

All patients consented and underwent a multislice computed tomography of the heart at baseline, 48 weeks, and 104 weeks to estimate the coronary artery calcium (CAC) score, aortic valve calcium score, and mitral annulus calcium score using the General Electric Revolution Evo scanner (GE Healthcare). Vessel score was the sum of all calcium scores of that vessel, and the CAC score was the sum of all calcium scores from the left main, left anterior descending, left circumflex, and right coronary arteries. The aortic valve calcium score was the sum of calcium scores of the aortic valve and aortic annulus. The mitral annulus calcium score was the sum of calcium scores of the mitral valve and mitral annulus (Item S1).

### Measurement of Aortic PWV

Aortic PWV, a measure of arterial stiffness, was evaluated by applanation tonometry of the carotid and femoral arteries, with the patient lying in the supine position, using the SphgymoCor device, version 8.0 (Atcor Medical), at the baseline, 48 weeks, and 104 weeks. PWV measurements with a standard deviation of less than 10% were used for the analysis. Two PWV measures that met the quality standard were averaged to give the final PWV reading (Item S1).

### Biochemical Measurements

Fifteen milliliters of fasting venous blood was collected from all patients who consented to the measurement of the serum T50, serum calcium, phosphorus alkaline phosphatase, iPTH, low-density lipoprotein (LDL) cholesterol, and C-reactive protein levels at the baseline. The serum calcium, phosphorus, and alkaline phosphatase levels were measured every 12 weeks throughout the study. The serum T50, iPTH, LDL cholesterol, and C-reactive protein levels were measured every 24 weeks. Samples of 24-hour urine and dialysate were collected at the baseline and then annually for the measurement of weekly urea clearance and weekly creatinine clearance. Normalized protein catabolic rate was estimated using the Randerson equation. All other laboratory tests were performed at a standard accredited university hospital biochemistry laboratory.

### Serum T50 Measurement

The T_50_ calcification propensity test has been described previously (Item S1).^13^

### Study Endpoints

The primary study endpoints were changes in the coronary CAC score and aortic PWV over 104 weeks. The secondary endpoints included changes in the aortic valve calcium score; mitral annulus calcium score; serum T50 level; biochemical parameters of CKD–MBD, including serum calcium, phosphorus, alkaline phosphatase, and iPTH levels; C-reactive protein level; and LDL cholesterol level over 104 weeks. As a secondary endpoint, we analyzed annualized percentage change in the CAC score over 104 weeks (Item S1). We defined rapid progressors as participants with an annualized percentage change in the CAC score of ≥15%. This percentage threshold has also been adopted as a clinically meaningful value for the progression of coronary artery calcification in other studies.^4^^,^^16^, ^17^, ^18^

### Statistical Analysis

Data were expressed as mean ± standard deviation or median (interquartile range), depending on the distribution. The normality of the data was tested using the Shapiro-Wilk test. A linear mixed-effect model analysis was used to compare changes over time between the 2 groups for the primary endpoints and various secondary endpoints. The within-group and between-group effect sizes were calculated using Cohen d. Data not normally distributed were log transformed before the linear mixed-effect model analysis. Between-group comparisons for nonparametric data were performed using the Mann-Whitney *U* test. A *P* value <0.05 was considered statistically significant. The statistical analysis was performed using SPSS, version 25.0 (SPSS, Inc).

## Results

**Figure 1:**
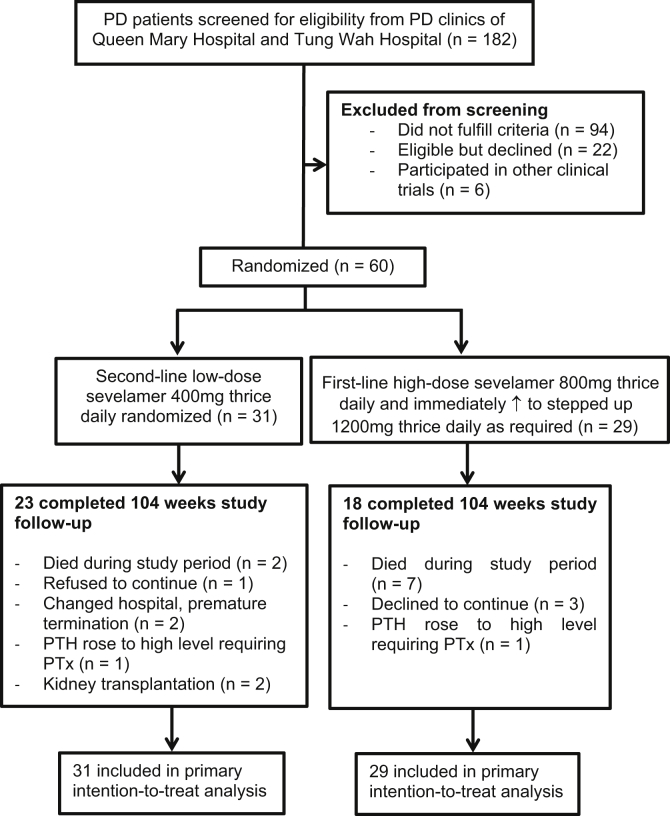
Study consort diagram. Abbreviations: PD, peritoneal dialysis; PTH, parathyroid hormone; PTx, parathyroidectomy.

**Figure 2:**
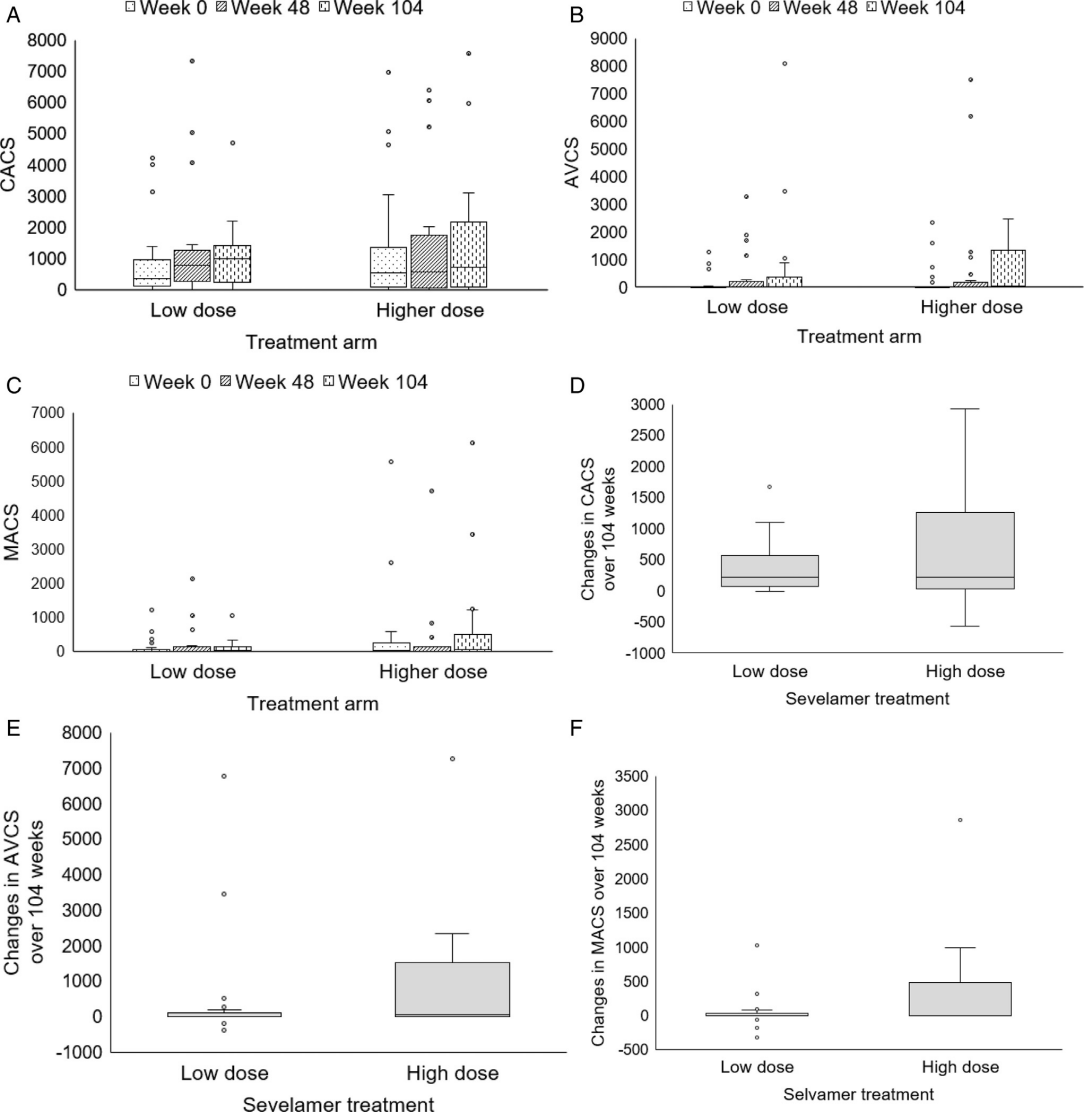

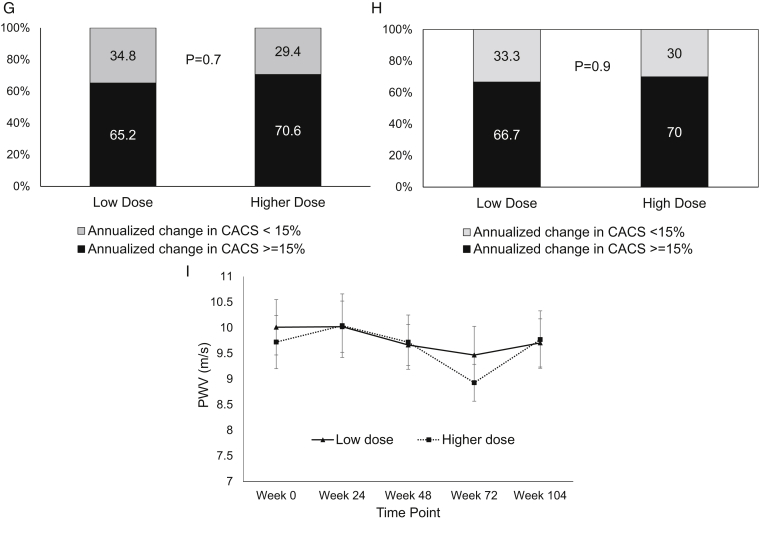
(A) CACS, (B) AVCS, and (C) MACS at the baseline, week 48, and week 104 in the low-dose and high-dose sevelamer treatment groups (median [interquartile range] in box plots) and within-group changes calculated using the Wilcoxon signed-rank test. Changes in (D) CACS, (E) AVCS, and (F) MACS over 104 weeks in the low-dose and high-dose sevelamer treatment groups (median [interquartile range] in box plots) and between-group differences calculated using the Mann-Whitney *U* test. (G) Proportion (%) of rapid progressors of coronary artery calcification in the low-dose and high-dose treatment groups. (H) Proportion (%) of rapid progressors of coronary artery calcification among those with a CACS of ≥100 at the time of study entry. (I) Serial aortic PWV (mean ± standard error) over 104 weeks in the 2 treatment groups. Abbreviations: AVCS, aortic valve calcium score; CACS, coronary artery calcium score; MACS, mitral annulus calcium score; PWV, pulse wave velocity.

Of 182 screened individuals, 60 patients receiving PD were enrolled in the study. Figure 1 depicts the study consort diagram. Table 1 presents the baseline characteristics. Table 2 presents the baseline CAC score, aortic valve calcium score, mitral annulus calcium score, aortic PWV, and serum T50 level. The serial CAC score, aortic valve calcium score, and mitral annulus calcium score (median [interquartile range]) over 104 weeks are depicted in Table 3 and Fig 2A-C. Changes in the CAC score, aortic valve calcium score, and mitral annulus calcium score over 104 weeks did not differ between the 2 groups (Fig 2D-F). The proportion of rapid progressors did not differ between the 2 groups (Fig 2G). Including only patients with a baseline CAC score of ≥100, the proportion of rapid progressors did not differ between the 2 groups (Fig 2H). Changes in aortic PWV over 104 weeks did not differ between the 2 groups (Table 3, Fig 2I). One patient in the high-dose sevelamer group failed the PWV assessment at weeks 72 and 104 because of new-onset persistent atrial fibrillation.

**Table 1:** Baseline Characteristics

Parameters	Second-Line, Low-Dose Sevelamer Group (n = 31)	First-Line, High-Dose Sevelamer Group (n = 29)
Demographic and clinical parameters		
Male:female	16:15	18:11
Age, y	53.3 ± 10.4	55.6 ± 12.5
Positive smoking history, n (%)		
Current smoker	2 (6.5)	2 (6.9)
Ex-smoker	7 (22.6)	4 (13.8)
Background diabetes, n (%)	8 (25.8)	9 (31)
Background coronary artery disease, n (%)	2 (6.5)	4 (13.8)
Background ischemic/hemorrhagic CVD, n (%)	2 (6.5)	1 (3.4)
Background heart failure, n (%)	3 (9.7)	6 (20.7)
Previous parathyroidectomy, n (%)	6 (19.4)	2 (6.9)
Charlson comorbid score	3.9 ± 2.0	4.1 ± 2.0
Duration of PD, mo^a^	51.2 (19.7-68.0)	39.2 (17.0-70.2)
Body mass index, kg/m^2^	24.1 ± 3.3	24.2 ± 3.4
Systolic blood pressure, mm Hg	128 ± 21	132 ± 22
Diastolic blood pressure, mm Hg	73 ± 11	77 ± 14
Dialysis parameters		
Total weekly Kt/V	1.92 ± 0.37	2.00 ± 0.24
Total weekly PD Kt/V	1.84 ± 0.42	1.87 ± 0.28
Total weekly CrCl, L/wk/1.73 m^2^^a^	48.1 (46.3-53.9)	50.7 (47.6-52.3)
Total weekly PD CrCl, L/wk/1.73 m^2^	43.6 ± 10.1	46.4 ± 8.2
Normalized PCR, g/kg/m^2^	0.99 ± 0.22	1.01 ± 0.19
N (%) with anuria	18 (58.1)	17 (58.6)
Residual GFR, mL/min/1.73 m^2^^a^	0 (0, 0.72)	0 (0, 1.44)
Dialysate to plasma creatinine ratio	0.64 ± 0.11	0.67 ± 0.13
Total daily PD exchanges volume, L	7.1 ± 1.7	7.7 ± 1.8
Use of low calcium (2.5 mEq/L) PD fluids, n (%)	31 (100)	27 (93.1)
Medications		
Use of vitamin D analogues, n (%)		
No use	11 (35.5)	14 (48.3)
Rocaltrol	14 (45.2)	10 (34.5)
Alfacalcidiol	6 (19.4)	5 (17.2)
Calcium-based phosphorus binders, n (%)	24 (77.4)	19 (65.5)
Aluminum-based phosphorus binders, n (%)	14 (45.2)	22 (75.9)
Total number of antihypertensives	2.65 ± 1.50	2.59 ± 1.45
Renin–angiotensin aldosterone blockers, n (%)	15 (48.4)	17 (58.6)
Calcium–channel blockers, n (%)	21 (67.7)	22 (75.9)
Beta blockers, n (%)	19 (61.3)	15 (51.7)
Statins or fibrates, n (%)	15 (48.4)	8 (27.6)
Aspirin, n (%)	5 (16.1)	5 (17.2)
Biochemical parameters		
Serum calcium, mg/dL	9.73 ± 0.50	9.79 ± 0.67
Serum phosphorus, mg/dL	6.55 ± 1.24	6.28 ± 1.27
Alkaline phosphatase, U/L^a^	83 (56-103)	87 (57-137)
iPTH, pg/mL	420 ± 292	374 ± 233
Serum T50, min	286 ± 84	266 ± 84
C-reactive protein, mg/L^a^	0.35 (0.35-0.89)	0.35 (0.35-0.61)
Serum albumin, g/dL	3.73 ± 0.37	3.68 ± 0.42
Fasting glucose, mg/dL	109 ± 7.15	111 ± 4.86
HDL cholesterol, mg/dL	44.0 ± 2.17	43.9 ± 2.99
LDL cholesterol, mg/dL	116 ± 40.1	97.2 ± 43.1
Triglyceride, mg/dL	172 ± 22.2	157 ± 18.4
Hemoglobin, g/dL	9.92 ± 1.53	9.67 ± 1.47
Hematocrit, %	29.3 ± 4.5	28.6 ± 4.5

*Note:* Continuous data are expressed as mean ± standard deviation, unless otherwise specified. Second-line, low-dose sevelamer was used in the intervention group; first-line, high-dose sevelamer was used in the control group. The conversion factors for the units are as follows: serum calcium in mg/dL to mmol/L, x 0.2495; serum phosphate in mg/dL to mmol/L, x 0.3229; iPTH in pg/mL to pmol/L, x 0.106; serum albumin in g/dL to g/L, x 10; fasting glucose from mg/dL to mmol/L, x 0.05551; HDL cholesterol and LDL cholesterol from mg/dL to mmol/L, x 0.02586; and triglyceride from mg/dL to mmol/L, x 0.01129.

Abbreviations: CrCl, creatinine clearance; CVD, cerebrovascular disease; GFR, glomerular filtration rate; HDL, high-density lipoprotein; iPTH, intact parathyroid hormone; Kt/V, urea clearance; LDL, low-density lipoprotein; PCR, protein catabolic rate; PD, peritoneal dialysis; T50, calcification propensity test.

a Median (interquartile range).

**Table 2:** CAC and Heart Valve Calcium Scores, Aortic PWV, and T50 of the Study Population at the Time of Study Entry

	Second-Line, Low-Dose Sevelamer Group (n = 31)	First-Line, High-Dose Sevelamer Group (n = 29)	*P* Value
CAC score^a^	330 (124-845)	603 (78-1,367)	0.32
Aortic valve calcium score^a^	0 (0-10)	0 (0-19)	0.89
Mitral annulus calcium score^a^	0 (0-23)	0 (0-309)	0.11
Aortic PWV, m/s	10.0 ± 3.0	9.7 ± 2.8	0.70
T50, min	285 ± 84	266 ± 84	0.39

*Note:* Data are expressed as mean ± standard deviation unless otherwise specified.

Abbreviations: CAC, coronary artery calcium; PWV, pulse wave velocity; T50, calcification propensity test.

a Expressed as median (interquartile range) in view of skewed distribution.

**Table 3:** Serial CAC Scores, Heart Valve Calcium Scores, Aortic PWV, and T50 Over 104 Weeks

	Low-Dose Sevelamer (n = 31)			High-Dose Sevelamer (n = 29)			Between-Group Effect Size^d^	*P* Value^e^
	Estimated Mean ± SE or Median (IQR)^a^^,^^b^	Within-Group Effect Size^c^	Change from baseline Mean ± SE or Median (IQR)^a^^,^^b^	Estimated Mean ± SE or Median (IQR)^a^^,^^b^	Within-Group Effect Size^c^	Change From Baseline Mean ± SE or Median (IQR)^a^^,^^b^		
CAC score^b^^,^^f^								
Baseline	330 (124-845)	—	—	603 (78-1,367)	—	—	—	—
Week 48	748 (231-1,176)	0.21	127 (18-276)	587 (71-1,970)	0.22	178 (45-756)	0.07	0.99
Week 104	1,000 (231-1,358)	0.35	225 (78.5-525)	714 (130-2,152)	0.58	223 (56-1,212)	0.11	0.21
Aortic valve calcium score^b^^,^^f^								
Baseline	0 (0-10)	—	—	0 (0-19)	—	—	—	—
Week 48	0 (0-35)	0.17	0 (0-5)	0 (0-240)	0.17	0 (0-238)	0.02	0.97
Week 104	0 (0-366)	0.29	0 (0-76.5)	55 (0-1,334)	0.81	53 (0-1,278)	0.47	0.10
Mitral annulus calcium score^b^^,^^f^								
Baseline	0 (0-22.5)	—	—	20 (0-273)	—	—	—	—
Week 48	8.5 (0-71)	0.18	0 (0-17)	6.5 (0-408)	0.02	0 (0-35)	0.16	0.71
Week 104	26 (0-100)	0.36	0 (0-30)	43 (0-491)	0.32	1 (0-405)	0.31	0.16
PWV, m/s								
Baseline	10.0 ± 0.5	—	—	9.7 ± 0.5	—	—	—	—
Week 24	10.1 ± 0.5	0.04	0.1 ± 0.1	10.0 ± 0.5	0.09	0.3 ± 0.1	0.06	0.81
Week 48	10.2 ± 0.5	0.07	0.2 ± 0.1	9.7 ± 0.5	0	0 ± 0.1	0.19	0.71
Week 72	10.0 ± 0.5	0.004	0 ± 0.1	9.8 ± 0.6	0.04	0.1 ± 0.2	0.15	0.86
Week 104	10.3 ± 0.5	0.09	0.3 ± 0.1	10.5 ± 0.6	0.31	0.8 ± 0.2	0.11	0.31
T50, min								
Baseline	285 ± 15	—	—	266 ± 16	—	—	—	—
Week 24	288 ± 17	0.02	3 ± 4.8	292 ± 18	0.31	26 ± 4.6	0.05	0.64
Week 48	298 ± 17	0.14	13 ± 4.9	248 ± 17	0.19	−18 ± 4.5	0.57	0.22
Week 72	288 ± 18	0.02	3 ± 5.1	231 ± 20	0.39	−35 ± 5.5	0.66	0.29
Week 104	296 ± 19	0.11	11 ± 5.6	260 ± 22	0.06	−6 ± 6.3	0.43	0.33

Abbreviations: CAC, coronary artery calcium; IQR, interquartile range; PWV, pulse wave velocity; SE, standard error; T50, transformation time of amorphous calcium phosphate-containing primary calciprotein particles to crystalline hydroxyapatite-containing secondary calciprotein particles.

a Estimated mean ± SE from the result of linear mixed-effects model.

b Data are presented as median (IQR) in view of skewed distribution.

c The effect size was calculated from the difference of estimated mean and standard deviation at each time point to baseline.

d The effect size was calculated with the between-group difference divided by the pooled standard deviation.

e The *P* value was assessed by the linear mixed-effect model.

f Data were log transformed (because of their skewed distribution) before linear mixed-effects model analysis.

**Figure 3:**
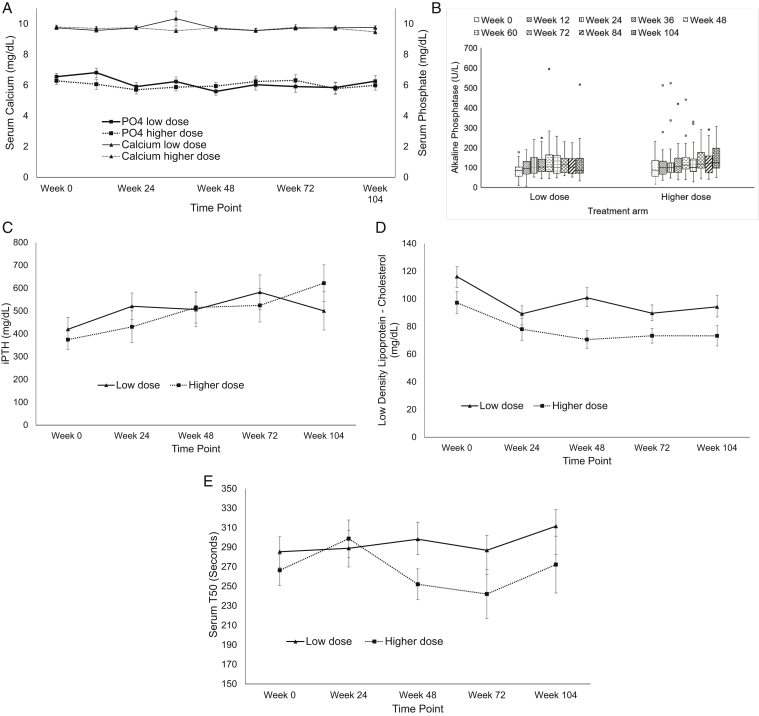
Serial (A) mean serum calcium and phosphorus, (B) alkaline phosphatase, (C) iPTH, (D) low-density lipoprotein cholesterol, and (E) serum T50 over 104 weeks in the low-dose and high-dose sevelamer treatment groups. Data are presented as mean ± standard error, except alkaline phosphatase, which is presented as median (interquartile range) in box plots. Abbreviations: iPTH; intact parathyroid hormone; PO4, phosphorus; T50, calcification propensity test.

Table S1 presents serial biochemical and hemodynamic parameters. The 2 groups did not significantly differ in the serum calcium and serum phosphorus levels over 104 weeks (Fig 3A). The serum alkaline phosphatase level increased over 104 weeks in both the groups, with no significant between-group difference (Fig 3B). The iPTH levels did not differ between the 2 groups, except at week 104, but became insignificant after Bonferroni correction (Fig 3C). The LDL cholesterol level decreased in both the groups over 104 weeks, with no significant between-group differences (Fig 3D). Serum T50 (Fig 3E) showed no significant within-group or between-group difference over 104 weeks. Table S2 details the dialysis parameters. The normalized protein catabolic rate showed no significant within-group or between-group difference over 104 weeks. Overall, the low-dose sevelamer group received a higher weekly median calcitriol dose but a lower elemental calcium dose than the high-dose sevelamer group (Table S3).

During the 104-week period, 9 patients (7 in the first-line, high-dose group and 2 in the second-line, low-dose group) died, 4 refused to continue the study, 2 underwent kidney transplants and dropped out, and 2 moved to follow-up at another hospital and dropped out. Two had their iPTH level increase to very high levels, requiring parathyroidectomy (1 in the high-dose and 1 in the low-dose group), and sevelamer was stopped after the parathyroidectomy because the serum phosphorus level normalized after the surgery. No patients received cinacalcet (Table S4). Detailed causes of the deaths are presented in Table S4. No death was deemed to be related to CKD–MBD treatment. Table S5 presents serious adverse events that resulted in hospitalizations. The high-dose group had more hospitalization episodes and more cardiovascular-related hospitalizations than the low-dose group.

## Discussion

The primary result showed that sevelamer used as a second-line, low-dose therapy with calcium carbonate showed similar progressions in the CAC score, aortic valve calcium score, and mitral annulus calcium score compared with sevelamer used as a first-line, high-dose therapy over 104 weeks. Although our results are in contrast with those of an earlier randomized study by Block et al,^4^ showing less progression in the CAC score with a sevelamer-based phosphorus binder, they add to the recent systematic review by the Cochrane group, which suggested no clinically important benefits of sevelamer on coronary artery calcification or cardiovascular outcomes based on clinical trials to date.^9^ In our study, the sevelamer dose used in the high-dose group was between 2,400 mg and 3, 600 mg per day. However, it did not stabilize or regress the CAC or heart valve calcium scores.

Notably, we did not observe progression in aortic PWV in both the groups. This raises the possibility that sevelamer treatment, irrespective of the dose used, may stabilize, although not reverse, arterial stiffness in patients receiving PD. Our findings are in keeping with those of the IMpact of Phosphate Reduction On Vascular End-points in Chronic Kidney Disease (IMPROVE)-CKD trial, which showed no significant changes in aortic PWV with phosphorus lowering using another non–calcium-based phosphorus binder.^19^ As the baseline CAC scores were very high and the vessels were already stiff, it might have been unlikely that any intervention would have made a large difference, particularly when the calcium balance between the groups was similar. The aortic PWV data of our Chinese patients receiving dialysis were comparable with those of the IMPROVE-CKD trial.^19^ Another previous study observed a significant increase in arterial stiffness over 1 year in patients with advanced CKD.^20^ Our findings are consistent with those of a randomized trial by Chue et al,^21^ which did not detect a significant change in aortic PWV with sevelamer compared with a placebo. However, the study by Chue et al^21^ was of a shorter duration, only 40 weeks, and the sevelamer dose used was 1,600 mg per day. This contrasts with the sevelamer dose of 2,400 to 3,600 mg per day that was used in the high-dose group in the current study.

In the current study, sevelamer treatment, whether at a low or high dose, did not differ in its effect on the serum calcium and phosphorus levels. According to Fig 3A, the mean serum phosphorus level was above the target of 5.51 mg/dL throughout 104 weeks in both the groups. Suboptimal phosphorus control and practical difficulties in achieving a serum phosphorus level below the study target may partly explain the increase in the CAC scores and heart valves calcium scores in the 2 groups. On the other hand, both the groups were able to maintain an average serum calcium level between 9.2 and 10 mg/dL, with no significant between-group difference. Notably, the alkaline phosphatase and iPTH levels increased in both the groups. This suggests that iPTH and alkaline phosphatase level increases are independent of sevelamer dose and more likely to be driven by suboptimal phosphorus control. Our results somewhat contrasted with those of another study showing that sevelamer reduced rather than increased the iPTH level in patients receiving PD, and this may be explained by concomitant treatment with a vitamin D analogue.^22^ In our study, despite increasing doses of a vitamin D analogue being prescribed, the iPTH and alkaline phosphatase levels continued to show gradual increases during the study period in both the groups.

Notably, serum T50 did not change in either treatment group. A previous study has shown that an increase in serum T50 may be associated with decreased arterial stiffening and lower mortality in patients with CKD.^14^ Possible explanations for the failure to increase T50 could be that the mean serum phosphorus level remained suboptimal in our patients receiving PD; the sevelamer dose used, even with the higher dose, might have been insufficient to increase serum T50 to a level that slows the progression of vascular and valvular calcification; or sevelamer did not decrease the C-reactive protein level in our patients. Further study is needed to evaluate what T50 level or threshold is required to inhibit vascular calcification.

It is currently uncertain if any phosphorus binder would affect hard outcomes compared with a placebo. Two pragmatic randomized trials that are currently underway to evaluate whether high versus standard or low phosphate targets affect survival and cardiovascular outcomes in patients receiving dialysis would shed more light on this question.^23^^,^^24^ Because of cost constraints, sevelamer-based phosphorus binders are not reimbursed in many middle-income or low-income countries, and many patients can not afford self-financed, long-term use of sevelamer. At the time of this study, emerging data suggested that restricting the dose of a calcium-based phosphorus binder in patients with kidney failure is associated with more favorable clinical outcomes compared with a high dose of a calcium-based phosphorus binder. If sevelamer used as a second-line, low-dose therapy (thus allowing a restricted dose of calcium-based phosphorus binders to be used to avoid hypercalcemia) is equally effective as a first-line, high-dose therapy in controlling the biochemical parameters of CKD–MBD and limiting vascular calcification and arterial stiffness, this could be cost saving for many emerging countries or countries that do not cover the drug cost.

In keeping with previous reports, sevelamer, whether at a low or high dose, had pleiotropic effects and significantly lowered the LDL cholesterol levels over 104 weeks.^22^ Both low-dose and high-dose sevelamer treatments were associated with an average of 25% to 30% reduction in the LDL cholesterol level. This suggests that sevelamer has potential cardiovascular protective benefits beyond phosphorus lowering or calcium sparing, which warrants further exploration.

Several limitations of this study are worth noting. First, this study was designed at the time to determine whether a low dose of sevelamer used as a second-line therapy with a calcium-based phosphorus binder was equally effective as a first-line therapy with a higher dose of sevelamer in controlling the biochemical parameters of CKD–MBD, limiting vascular calcification and arterial stiffness. A third group in which a calcium-based phosphorus binder alone was used was not available for comparison. Second, because the study was intended to be a pilot study at the time, we did not perform a sample size calculation. The sample size was small, with significant dropouts, and may not be powered enough to detect a statistically significant difference between the 2 groups. In addition, it is difficult to dissect the complex relationships between phosphorus control, calcium balance, and vitamin D analogue doses in predicting the progression of vascular or valvular calcification. Third, the study was open labeled because double blinding was difficult with different phosphorus binders and doses being used. Fourth, the study recruited prevalent patients receiving PD. Fifth, we did not do pill counts to check medication adherence. Sixth, 1 patient in each group underwent a parathyroidectomy, which has a major impact on bone mineral balance and may require a higher dose of calcitriol treatment that could possibly impact outcomes. Finally, a cost-effectiveness analysis was not included at the time of the trial design.

However, our study has the following strengths. First, the rationale of the study and the practice adopted are well in keeping with the latest Kidney Disease Improving Global Outcomes 2017 CKD–MBD Clinical Practice Guidelines in several aspects: avoid hypercalcemia recommendation grading (2C), restrict the dose of a calcium-based phosphorus binder (2B), and avoid the long-term use of an aluminum-based phosphorus binder (1C).^25^ Our findings are, therefore, applicable and relevant for real clinical practice, especially in countries with limited health resources and cost constraints that may prohibit the first-line use of high-dose sevelamer. Our findings lend important support to the approach that a low dose of sevelamer hydrochloride at 400 mg thrice daily may be introduced as a second-line therapy for hyperphosphatemia after a calcium-based phosphorus binder is used and the serum calcium level has reached the upper limit of the laboratory reference to avoid hypercalcemia. In this way, more patients may benefit from using sevelamer without adding a significant burden to health care expenditure, and this may facilitate better implementation of the Kidney Disease Improving Global Outcomes 2017 CKD–MBD guideline update.^26^ Second, our study is, to our knowledge, the first to evaluate the long-term safety and efficacy of a sevelamer-based phosphorus binder in controlling CKD–MBD, vascular or valvular calcification, and arterial stiffening in patients receiving PD. Our data should be applicable and generalizable to other PD populations worldwide.

In conclusion, sevelamer used as a second-line, low-dose therapy in combination with calcium carbonate had similar effects on coronary arteries, aortic valve and mitral annulus calcification, aortic stiffness, T50, and biochemical parameters of CKD–MBD over 2 years as first-line, high-dose sevelamer therapy in patients receiving PD. A low dose of sevelamer may be adopted as a second-line therapy for hyperphosphatemia in patients receiving PD in resource-constrained countries to minimize calcium loading.

## Supplementary Material

Supplementary File (PDF) Item S1; Table S1-S5.
