# Supplementary material for: Long-Term Effects of Sevelamer on Vascular Calcification, Arterial Stiffness, and Calcification Propensity in Patients Receiving Peritoneal Dialysis: The Randomized Pilot SERENE (Sevelamer on Vascular Calcification, Arterial Stiffness) Trial
Source: Kidney Med. 2021 Nov 2;4(2):100384. doi: 10.1016/j.xkme.2021.10.002 (PMC8861951; doi:10.1016/j.xkme.2021.10.002)
Supplement: Supplementary File (PDF) — Item S1; Table S1-S5. [file mmc1.pdf]

Item S1. Additional Methods

Multi-slice Computed Tomography (MSCT) of Coronary Arteries and Heart Valve

Scans were obtained with 120 kV, 400 - 700 mA (depending on patient size), and 0.35 sec rotation scanning time, with electrocardiography (ECG) triggering of images at 80% of the R-R interval. Data was acquired by conventional transverse scanning in contiguous 2.5 mm sections, from - 2cm below carina to the base of the heart. The sequence required an approximately 30 second breath hold to completely interrogate the heart. Scanning data was transferred electronically to a workstation and calcium scores analyses were carried out using the software package on the GE Advantage Window workstation (version 4.7). The total Agatston-Janowitz score was used as the scoring method of the MSCT images of the entire coronary arterial tree from base to apex. To be measurable in the Agatston-Janowitz score, calcium plaque must reach at least 1 mm^2-with > 130 Hounsfield units (HU) or ≥ 3 adjacent pixels. Calcifications that were lower in attenuation were not included in the score. The score of each calcification was calculated by multiplying area of calcified plaque by an attenuation-weighting factor based on the highest HU value of calcified plaque [22]. If the lesion attenuation was 130–199 HU, the area score was multiplied by one; if it was 200–399, it was multiplied by two; if it was 300–399, it was multiplied by three; and if it was ≥ 400, it was multiplied by four [22].

Measurement of Aortic PWV

Pulse wave signals were captured non-invasively using an arterial tonometer placed over the carotid and femoral artery sequentially with simultaneous ECG recordings. A minimum of 12 seconds with approximately 10 pulse wave signals were recorded after a strong and reproducible pulse wave signal was obtained. The distance from the carotid to femoral artery was measured directly between each artery location and the supra-sternal

notch and the measurements were inputted into SphygmoCor software. PWV was calculated by measuring the time delay between two characteristic timing points on two pressure waveforms that were at a known distance apart and was automatically calculated by Atcor software.

Serum T50 Measurement

For the present study, T50 was performed with minor changes that did not materially change the test when compared with the original description. Serum samples were measured in triplicate in 384-well plates at 37°C over 600 minutes in a Nephelostar nephelometer (BMG Labtech, Ortenberg, Germany) and in a blinded manner. Stock solutions were a calcium and a phosphate solution. The pH was adjusted to 7.40 at 37°C in both solutions. For measurement, 35 ml of calcium solution was mixed with 40 ml serum, and then 25 ml of the phosphate solution was added. Data analyses of nonlinear regression curves were performed using Microsoft Excel software to determine the half-maximal precipitation time (T50). The analytical coefficients of variation of standards precipitating at 120, 260, and 390 minutes were 7.8%, 5.1%, and 5.9%, respectively. Serum samples stored at minus 80° and never thawed was shipped as a batch in dry ice and have T50 measured in Dr. Pasch's laboratory in Bern, Switzerland.

Annualized percentage change in CACS over 104 weeks

We first estimated percentage change in CACS scores over 104 weeks as follows: (CACS at week 104 – CACS at study entry)*100/(CACS at study entry + 1). Adding 1 to the CACS at study entry in the denominator enabled the analysis of patients with CACS of 0. Annualized percentage change in CACS score was then estimated by multiplying the above percentage change by 12/24.

Table S1. Serial biochemical and hemodynamic parameters of the two groups

	Sevelamer low dose (n=31)			Sevelamer higher dose (n=29)				
	Estimated Mean ± SE[a] or Median (IQR)[f]	Within Group Effect Size[b]	Change from baseline Mean ± SE[a] or Median (IQR)[f]	Estimated Mean ± SE[a] or Median (IQR)[f]	Within Group Effect Size[b]	Change from baseline Mean ± SE[a] or Median (IQR)[f]	Between Group Effect Size[c]	*P*-value[d]
**iPTH, pg/mL**								
Baseline	420 ± 63			374 ± 65				
Week 42	539 ± 64	0.34	119 ± 17	434 ± 67	0.16	60 ± 17	0.30	0.38
Week 48	538 ± 66	0.34	118 ± 18	518 ± 67	0.41	144 ± 17	0.06	0.76
Week 72	607 ± 66	0.54	187 ± 18	565 ± 70	0.56	191 ± 18	0.12	0.97
Week 104	541 ± 68	0.35	121 ± 19	698 ± 73	0.95	324 ± 19	0.48	0.04
**Serum calcium, mg/dL**								
Baseline	9.73 ± 0.09			9.79 ± 0.13				
Week 24	9.73 ± 0.12	0	0 ± 0.03	9.74 ± 0.13	0.07	-0.05 ± 0.03	0.02	0.83
Week 48	9.67 ± 0.13	0.09	-0.06 ± 0.03	9.74 ± 0.13	0.07	-0.05 ± 0.03	0.10	0.90
Week 72	9.70 ± 0.13	0.04	-0.03 ± 0.03	9.78 ± 0.14	0.01	-0.01 ± 0.04	0.12	0.94
Week 104	9.74 ± 0.14	0.01	0.01 ± 0.04	9.56 ± 0.15	0.33	-0.23 ± 0.04	0.26	0.32
**Serum phosphorus, mg/dL**								
Baseline	6.55 ± 0.22			6.28 ± 0.24				
Week 24	5.94 ± 0.27	0.42	-0.61 ± 0.07	5.70 ± 0.28	0.40	-0.58 ± 0.07	0.16	0.92
Week 48	5.63 ± 0.27	0.64	-0.92 ± 0.07	5.94 ± 0.28	0.23	-0.34 ± 0.07	0.22	0.18
Week 72	5.89 ± 0.28	0.45	-0.66 ± 0.07	6.28 ± 0.29	0	0 ± 0.08	0.27	0.23
Week 104	6.25 ± 0.29	0.21	-0.3 ± 0.08	5.93 ± 0.32	0.24	-0.35 ± 0.08	0.22	0.96

**ALP, U/L[e, f]**								
Baseline	83 (56, 103)			87 (57, 137)				
Week 24	101 (72, 152)	0.68	27 (7, 48)	99 (75, 123)	0.42	9 (-4, 25)	0	0.30
Week 48	108 (80, 164)	0.97	31 (12.5, 85)	111 (92, 151)	0.66	25 (8.5, 42.5)	0.05	0.25
Week 72	115 (73, 147)	0.83	35 (13, 65)	116 (96, 174)	0.70	28 (9.5, 51.5)	0.18	0.70
Week 104	84 (71, 148)	0.60	21 (-10.5, 57)	123 (97, 196)	0.96	43 (10, 66)	0.55	0.45
**Albumin, g/dL**								
Baseline	3.73 ± 0.08			3.68 ± 0.08				
Week 24	3.74 ± 0.08	0.02	0.01 ± 0.02	3.67 ± 0.08	0.02	-0.01 ± 0.02	0.16	0.85
Week 48	3.66 ± 0.08	0.16	-0.07 ± 0.02	3.81 ± 0.08	0.31	0.13 ± 0.02	0.36	0.12
Week 72	3.66 ± 0.08	0.16	-0.07 ± 0.02	3.76 ± 0.09	0.18	0.08 ± 0.02	0.23	0.27
Week 104	3.66 ± 0.09	0.16	-0.07 ± 0.03	3.60 ± 0.09	0.19	-0.08 ± 0.03	0.14	0.93
**LDL-Cholesterol, mg/dL**								
Baseline	116 ± 7			97 ± 7				
Week 24	89 ± 7	0.72	-27 ± 2	77 ± 7	0.55	-20 ± 2	0.34	0.32
Week 48	101 ± 7	0.41	-15 ± 2	70 ± 7	0.74	-27 ± 2	0.86	0.15
Week 72	92 ± 7	0.65	-24 ± 2	71 ± 7	0.72	-26 ± 2	0.60	0.79
Week 104	93 ± 7	0.65	-23 ± 2	70 ± 8	0.77	-27 ± 2	0.66	0.70
**Systolic BP, mmHg**								
Baseline	128 ± 4			132 ± 4				
Week 24	129 ± 4	0.04	1 ± 1	132 ± 4	0	0 ± 1	0.14	0.86
Week 48	129 ± 4	0.05	1 ± 1	128 ± 4	0.19	-4 ± 1	0.05	0.46
Week 72	125 ± 4	0.14	-3 ± 1	129 ± 4	0.15	-3 ± 1	0.19	0.99
Week 104	133 ± 4	0.24	5 ± 1	131 ± 5	0.05	-1 ± 1	0.10	0.48

**Diastolic BP, mmHg**								
Baseline	73 ± 2			77 ± 2				
Week 24	78 ± 2	0.35	5 ± 1	77 ± 2	0.02	0 ± 1	0.04	0.21
Week 48	73 ± 2	0.03	0 ± 1	76 ± 2	0.04	-1 ± 1	0.28	0.969
Week 72	74 ± 3	0.07	1 ± 1	77 ± 3	0.03	0 ± 1	0.19	0.70
Week 104	78 ± 3	0.39	5 ± 1	73 ± 3	0.35	-4 ± 1	0.46	0.01
**C-Reactive Protein[f], mg/L**								
Baseline	0.35 (0.35, 0.86)			0.35 (0.35, 0.57)				
Week 24	0.35 (0.35, 0.88)	0.09	0 (-0.01, 0)	0.35 (0.35, 0.73)	0.10	0 (0, 0.17)	0.05	0.98
Week 48	0.35 (0.35, 0.50)	0.13	0 (-0.08, 0)	0.35 (0.35, 0.55)	0.03	0 (-0.2, 0.01)	0.16	0.73
Week 72	0.35 (0.35, 0.84)	0.25	0 (0, 0.05)	0.35 (0.35, 1.07)	0.28	0 (0, 0.37)	0.01	0.88
Week 104	0.35 (0.35, 0.58)	0.07	0 (-0.05, 0.01)	0.35 (0.35, 0.58)	0.35	0 (-0.16, 0)	0.23	0.44

iPTH, intact parathyroid hormone; ALP, alkaline phosphatase; LDL-, low density lipoprotein-; BP, blood pressure.
[a]Estimated mean ± Standard Error (SE) from the result of liner mixed-effects model.
[b]Effect size was calculated from the difference of estimated mean and standard deviation at each time-point to baseline.
[c]Effect size was calculated from the between-group difference divided by pooled standard deviation.
[d]The p-value was assessed by the linear mixed-effect model.
[e]Data were log-transformed (due to their skewed distribution) before linear mixed-effects model analysis.
[f]Data expressed as median (interquartile range, IQR) in view of skewed distribution.

Note: Conversion factors for the units: Serum calcium in mg/dL to mmol/L, x 0.2495; Serum phosphorus in mg/dL to mmol/L, x 0.3229; iPTH in pg/mL to pmol/L, x 0.106; Serum albumin in g/dL to g/L, x 10; Fasting glucose from mg/dL to mmol/L, x 0.05551; HDL-cholesterol and LDL-cholesterol from mg/dL to mmol/L, x 0.02586; Triglyceride from mg/dL to mmol/L, x 0.01129.

Table S2. Serial dialysis parameters of the two groups

	Sevelamer low dose (n=31)			Sevelamer higher dose (n=29)				
	Estimated Mean ± SE[a] or Median (IQR)[f]	Within Group Effect Size[b]	Change from baseline Mean ± SE[a] or Median (IQR)[f]	Estimated Mean ± SE[a] or Median (IQR)[f]	Within Group Effect Size[b]	Change from baseline Mean ± SE[a] or Median (IQR)[f]	Between Group Effect Size[c]	*P*-value[d]
**Total Weekly Kt/V**								
Baseline	1.92 ± 0.05			2.00 ± 0.05				
Week 48	2.07 ± 0.06	0.52	0.15 ± 0.02	2.04 ± 0.06	0.14	0.04 ± 0.01	0.10	0.25
Week 104	2.02 ± 0.06	0.37	0.1 ± 0.02	2.06 ± 0.07	0.22	0.06 ± 0.02	0.15	0.64
**Weekly PD Kt/V**								
Baseline	1.84 ± 0.06			1.86 ± 0.06				
Week 48	2.01 ± 0.06	0.53	0.17 ± 0.06	1.97 ± 0.07	0.33	0.11 ± 0.02	0.15	0.93
Week 104	2.00 ± 0.07	0.49	0.16 ± 0.02	2.01 ± 0.08	0.48	0.15 ± 0.02	0.03	0.59
**Total Weekly CrCl[e], L/wk per 1.73m^2**								
Baseline	48.1 (46.3, 53.9)			50.7 (47.6, 62.3)				
Week 48	49.7 (45.4, 56.0)	0.28	3.6 (-3.3, 9.3)	50.3 (45.1, 56.6)	0.29	-2.9 (-4.9, 2.4)	0.10	0.01
Week 104	49.8 (40.3, 56.8)	0.10	1.2 (-6.1, 7.5)	50.2 (41.7, 54.4)	0.41	-3.5 (-9.1, 1.6)	0	0.04
**Weekly PD CrCl, L/wk per 1.73m^2**								
Baseline	43.6 ± 1.56			46.4 ± 1.61				
Week 48	47.6 ± 1.67	0.46	4 ± 0.4	47.3 ± 1.73	0.10	0.9 ± 0.4	0.04	0.20
Week 104	48.7 ± 1.89	0.60	5.1 ± 0.5	48.0 ± 2.13	0.19	1.6 ± 0.6	0.09	0.24
**nPCR, g/kg/day**								
Baseline	0.99 ± 0.05			1.02 ± 0.04				
Week 48	1.00 ± 0.04	0.04	0.01 ± 0.01	1.00 ± 0.04	0.10	-0.02 ± 0.01	0	0.61
Week 104	0.99 ± 0.04	0	0 ± 0.01	1.03 ± 0.05	0.05	0.01 ± 0.01	0.22	0.77

Kt/V, urea clearance; PD, peritoneal dialysis; CrCl, creatinine clearance; nPCR, normalized protein catabolic rate.

[a]Estimated mean ± Standard Error (SE) from the results of linear mixed-effects model.
[b]Effect size was calculated from the difference of estimated mean and standard deviation at each time-point to baseline.
[c]Effect size was calculated with the between-group difference divided by pooled standard deviation.
[d]The P-value for between-group difference was calculated by the linear mixed-effect model analysis.
[e]Variables were log-transformed (due to their skewed distribution) before linear mixed-effect model analysis.
[f]Data expressed as median (interquartile range, IQR) in view of skewed distribution.

Table S3. Daily elemental sevelamer dose and weekly vitamin D analog dose of the two treatment groups.

		Baseline	24 week	48 week	72 week	104 week
Elemental calcium dose (mg/day)	Low dose (n=31)	1000 (0, 1000)	1000 (0, 1000)	1000 (0, 1150)	800 (400, 1500)	250 (0, 1000)
	Higher dose (n=29)	500 (0, 1500)	1000 (0, 1500)	1000 (0, 1500)	1000 (0, 1500)	500 (0, 1500)
Sevelamer dose (mg/day)	Low dose (n=31)	1200 (1200, 1200)	1200, (1200, 1200)	1200, (1200, 1200)	1200, (1200, 1200)	1200, (1200, 1200)
	Higher dose (n=29)	2400 (2400, 2400)	2400 (2400, 2400)	2400 (2400, 2400)	3600 (2400, 3600)	3600 (2400, 3600)
Rocaltrol/alfacalcidol dose (µg/week)	Low dose (n=31)	0.50 (0, 1.50)	0.50 (0, 1.50)	0.88 (0.63, 1.69)	1.13 (0, 1.75)	1.00 (0, 1.75)
	Higher dose (n=29)	0.25 (0, 0.50)	0.50 (0, 1.50)	0.50 (0, 1.00)	0.75 (0.50, 1.50)	0.75 (0.18, 1.56)

Data presented as median (interquartile range, IQR).

Table S4. Outcomes of the two groups.

	Total (n=60)	Second-line Low dose group (n=31)	First-line higher dose group (n=29)	P-value*
Died during study	9 (15)	2 (6.5)	7 (24.1)	0.076
Breakdown causes of death				
- Peritonitis	2 (22.2)	0 (0)	2 (28.5)	
- Pneumonia	1 (11.1)	0 (0)	1 (14.3)	
- Sepsis	1 (11.1)	0 (0)	1 (14.3)	
- per rectal bleeding with hypotension	1 (11.1)	0 (0)	1 (14.3)	
- Intracerebral hemorrhage	1 (11.1)	0 (0)	1 (14.3)	
- Liver failure	1 (11.1)	0 (0)	1 (14.3)	
- Fluid overload and pneumonia	1 (11.1)	1 (50)	0 (0)	
- Metastatic malignancy	1 (11.1)	1 (50)	0 (0)	
Refused to continue	4 (6.7)	1 (3.2)	3 (10.3)	
Changed hospital, premature termination	2 (3.3)	2 (6.5)	0 (0)	
Underwent kidney transplantation	2 (3.3)	2 (6.5)	0 (0)	
iPTH rose to high level requiring PTx and no longer require sevelamer after PTx	2 (3.3)	1 (3.2)	1 (3.4)	

PTx, parathyroidectomy; iPTH, intact parathyroid hormone

*Fisher exact test.

Expressed as N (%).

Table S5. Hospitalizations of the two groups.

	Second-line low dose group (n=31)	First-line higher dose group (n=29)
Patients with no hospitalizations	10 (32.3)	10 (34.5)
Patients with hospitalizations	21 (67.7)	19 (65.5)
Total episodes of hospitalizations	44	60
Cardiovascular-related hospitalization episodes	8 (18.2)	21 (35)
-Acute myocardial infarction	2 (4.5)	2 (3.3)
-Heart failure/volume overload	1 (2.3)	11 (18.4)
-Peripheral vascular disease	1 (2.3)	2 (3.3)
-Arrhythmia	2 (4.5)	5 (8.3)
Atrial fibrillation	1	4
Sick Sinus syndrome	1	0
Third degree heart block	0	1
-Intracerebral hemorrhagic Stroke	1 (2.3)	1 (1.7)
-Poor blood pressure control	1 (2.3)	0 (0)
Non-cardiovascular related hospitalization episodes	36 (81.8)	36 (60)
-Peritonitis	12 (27.3)	11 (18.3)
-Infections other than peritonitis	10 (22.7)	16 (26.6)
-Hypotension	3 (6.8)	1 (1.7)
-Abdominal pain but not peritonitis	0 (0)	3 (5)
-Hypocalcemia	0 (0)	1 (1.7)
-Vasovagal syncope	1 (2.3)	0 (0)
-Malignancy-related	1 (2.3)	0 (0)
-Dehydration	1 (2.3)	0 (0)
-Intestinal obstruction	0 (0)	1 (1.7)
-Vertigo or dizziness	3 (6.8)	0 (0)
-Foot ulcer pain	1 (2.3)	0 (0)
-Nausea and vomiting	1 (2.3)	0 (0)
-Numbness	1 (2.3)	0 (0)
-Anemia	1 (2.3)	0 (0)
-Per rectal bleeding	1 (2.3)	1 (1.7)
-Scrotal swelling or hernia	0 (0)	2 (3.3)

Both cardiovascular and non-cardiovascular related in same hospitalization episode	0 (0)	3 (5)
-Infection and fluid overload	0 (0)	3 (5)

Expressed in N (%)
